# Incidence of Late-Onset Psoriasis Following Tonsillectomy: A Longitudinal Follow-Up Study Using a National Health Screening Cohort

**DOI:** 10.3390/jpm14060605

**Published:** 2024-06-06

**Authors:** Sung Joon Park, Hahn Jin Jung, Min Woo Park, Hyo Geun Choi, Heejin Kim, Jee Hye Wee

**Affiliations:** 1 Department of Otorhinolaryngology-Head and Neck Surgery, Chung-Ang University Gwangmyeong Hospital, Chung-Ang University College of Medicine, Seoul 14353, Republic of Korea; hypo23hns@cau.ac.kr; 2 Department of Otorhinolaryngology-Head and Neck Surgery, Chungbuk National University College of Medicine, Chungbuk National University Hospital, Cheongju 28644, Republic of Korea; hahnjin2@cbnu.ac.kr; 3 Department of Otorhinolaryngology-Head and Neck Surgery, Kangdong Sacred Heart Hospital, Seoul 05355, Republic of Korea; subintern@kdh.or.kr; 4 Department of Otorhinolaryngology-Head and Neck Surgery, Mdanalytics, Suseoseoulent Clinic, Seoul 06349, Republic of Korea; pupen@naver.com; 5 Department of Otorhinolaryngology-Head and Neck Surgery, Hallym University Sacred Heart Hospital, Hallym University College of Medicine, Anyang 14068, Republic of Korea; mir5020@hallym.or.kr

**Keywords:** tonsillectomy, psoriasis, cohort studies

## Abstract

Tonsillectomy has been suggested as a potential intervention to resolve psoriasis; however, its preventive effects on the development of psoriasis remain unclear. This study aimed to investigate the risk of developing late-onset psoriasis among a Korean adult population who had undergone tonsillectomy. Data from the Korean National Health Insurance Service-Health Screening Cohort between 2002 and 2019 were utilized. Out of a total of 514,866 participants, 1082 participants aged 40 years or older who had undergone tonsillectomy were matched with 4328 control participants using overlap weighting adjustment based on the propensity score. The incidence and hazard ratio (HR) of psoriasis were calculated for both tonsillectomy and control groups. The incidence rates of psoriasis were 1.30% in the tonsillectomy group and 1.20% in the control group. The incidence of psoriasis (overlap-weighted HR = 1.08, 95% confidence of interval = 0.69–1.69, and *p* = 0.732) did not differ significantly between the patients who underwent tonsillectomy and those in the control group. The cumulative probability of developing psoriasis was not different between the two groups (Log-rank test: *p* = 0.440). These findings were consistent across subgroups divided by age, sex, income, and region of residence. We found that tonsillectomy did not confer a preventive effect on the development of late-onset psoriasis in the Korean adult population.

## 1. Introduction

Psoriasis is one of the most common chronic inflammatory skin disorders, affecting 2% to 3% of the global population [1]. Recent estimates indicate a substantial global burden with 4,622,594 new cases, 40,805,386 existing cases, and a contribution of 3,505,736 disability-adjusted life years (DALYs) due to psoriasis [2]. According to a population-based study utilizing the Korean National Health Insurance Database, the standardized prevalence of psoriasis was 453 per 100,000 individuals in 2015 [3]. Various standard therapeutic approaches have been employed to manage psoriasis over periods. These include corticosteroids, methotrexate, cyclosporine, acitretin, and phototherapy [4]. The preferred treatment for patients with mild psoriasis is local topical treatment and systemic treatment is commonly used for severe disease. However, complete recovery from psoriasis using these treatments has not been documented.

It is widely recognized that psoriatic lesions result from the aberrant reactivity of specific T lymphocytes in the skin. However, the etiopathogenesis of psoriasis is known to be multi-factorial, and several theories have been advocated to explain the mechanism [5]. First, a chronic inflammatory response characterized as the autoimmunity of the host caused by activation of autoreactive T lymphocytes for over-expressed antimicrobial peptides, which serve as autoantigens, has been proposed. Second, identification of a loss of diversity and the pathogenic bacteria and the virus in psoriatic plaques have implicated that dysbiosis of skin microbiota has relevance in pathogenesis of psoriasis. Third, the higher release of reactive oxygen species and the disparity of oxidant and antioxidant mechanisms causing cell injury, which leads to the release of proinflammatory mediators and antimicrobial peptides, resulting in activation of the Th1/Th17 axis, have been hypothesized as a potential mechanism of psoriasis development.

In addition, the pathogenesis of psoriasis is strongly influenced by genetic predispositions and environmental triggers such as infection, stress, injuries, smoking, obesity, and medications. A major genetic susceptibility factor for psoriasis are the HLA-Cw6, HLA-Cw1, HLA-Cw12, HLA-B27, and HLA-DR*07 alleles [6]. Moreover, streptococcal throat infections have been identified as significant triggers for psoriasis in susceptible individuals [7]. Given this background, tonsillectomy has been proposed as a therapeutic intervention to mitigate psoriasis [8]. Patients with psoriasis frequently report sore throats more often than those without the condition [9]. It is hypothesized that a pathogenic streptococcal trigger within the palatine tonsils may activate skin-homing T cells in psoriasis through a mechanism of molecular mimicry. Previous studies have indicated that immunoglobulin (Ig) A levels against *Streptococcal pyogenes* are elevated in the plasma of patients with psoriasis compared to control subjects [7]. A comprehensive systematic review revealed that out of 410 psoriasis patients who underwent tonsillectomy, 290 experienced improvements in their psoriasis [10]. Some literature has suggested that in specific subtypes like guttate and plaque psoriasis, which are often triggered by streptococcal infections, tonsillectomy might reduce flare-ups. A Cochrane review including one randomized controlled trial evaluating tonsillectomy versus no treatment for chronic plaque psoriasis reported a relative risk (RR) of 6.56 for tonsillectomy in achieving a Psoriasis Area and Severity Index (PASI) 75 [11]. It indicated that tonsillectomy may have a beneficial effect on chronic plaque psoriasis because the palatine tonsils generate effector T cells that recognize keratin determinants in the skin. However, the preventive potential of tonsillectomy on psoriasis remains unclear.

Globally, the number of prevalent cases of psoriasis in 2019 reached 40.8 million, marking a 29.1% increase since 1990. This rise has been attributed to population growth (42.5%) and aging (18.3%) [12]. Studies that reported age-specific incidence rates have identified a dual peak in psoriasis prevalence around the age of 30–39 years and a second peak around 50–59 or 60–69 years [13]. This bimodal distribution of psoriasis incidence suggests two distinct clinical presentations of the disease: the “early-onset or inheritable type” and the “late-onset or noninheritable type”, defined as presenting at ≤40 and >40 years of age, respectively [14]. Therefore, this study focuses on evaluating the preventive effect of tonsillectomy on late-onset psoriasis in individuals over 40 years of age, aiming to elucidate the relationship between infectious triggers and psoriasis while excluding genetic factors. The aim of the study was to evaluate the risk of late-onset psoriasis (>40 years of age) in the general population who underwent tonsillectomy.

## 2. Materials and Methods

### 2.1. Ethics

The ethics committee of Hallym University (2019-10-023) authorized this study. Written informed consent was waived by the Institutional Review Board. All analyses were conducted in compliance with the guidelines and regulations established by the ethics committee of Hallym University.

### 2.2. Study Population and Participant Selection

**Figure 1:**
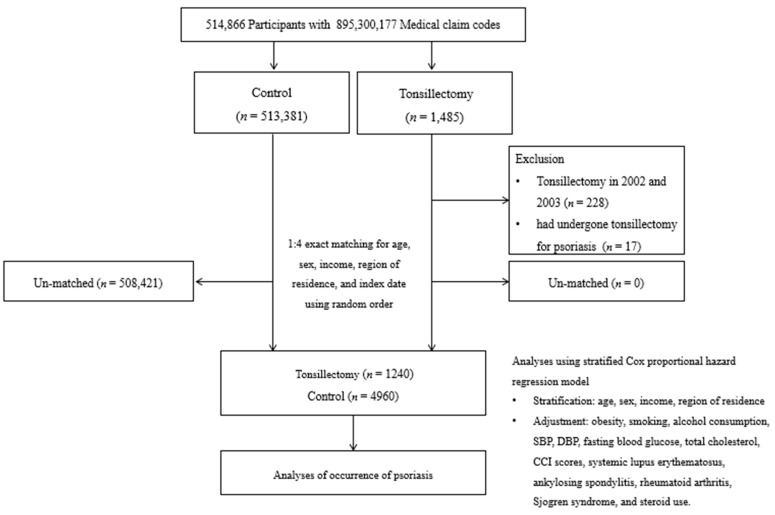
A schematic illustration of the participant selection process that was used in the present study. Of a total of 514,866 participants, 1240 tonsillectomy participants were matched with 4960 of control participants for age, sex, income, and region of residence.

The Korean National Health Insurance Service (NHIS)-Health Screening Cohort data, previously described in detail elsewhere [15], provided the basis for participant selection. The Korean National Health Insurance Service (NHIS) selects approximately 10% of random samples (*n* = 514,866) from all individuals who underwent health examinations between 2002 and 2003 (*n* ≈ 5,150,000). The age- and sex-specific distributions of the cohort population are detailed online. All Koreans aged 40 and older, along with their families, are encouraged to undergo biannual health checks at no cost. As all Korean citizens are assigned a unique 13-digit resident registration number that remains with them for life, comprehensive population statistics can be accurately compiled in this study. Registration with the NHIS is mandatory for all Koreans. This 13-digit number is required for all medical transactions in Korean hospitals and clinics, ensuring that medical records remain unique and preventing duplication, even if a patient relocates. Furthermore, the Korean Health Insurance Review and Assessment (HIRA) system oversees all medical treatments within the country. Causes and dates of death diagnosed by medical doctors are legally reported to the administrative entity via death certificates. The NHIS database includes health insurance claim codes (covering procedures and prescriptions), diagnostic codes based on the International Classification of Diseases-10th Revision (ICD-10), death records, socioeconomic data, and health check-up data. These health check-up data encompass body mass index (BMI), alcohol consumption, smoking habits, blood pressure, urinalysis, hemoglobin, fasting glucose, lipid profiles, creatinine, and liver enzymes for each participant from 2002 to 2013 [16]. From a pool of 514,866 individuals with 895,300,177 medical claim codes spanning from 2002 to 2019, 1485 participants who had undergone tonsillectomy were identified. The control group comprised individuals who had not undergone tonsillectomy during the same period (*n* = 513,381). To ensure cleanliness of the data, participants who underwent tonsillectomy from 2002 to 2003 were excluded to account for washout periods (*n* = 228), as well as those who had a tonsillectomy due to psoriasis (*n* = 17). Tonsillectomy participants were matched in a 1:4 ratio with control participants based on age, sex, income, and region of residence. Control participants were randomized to minimize selection bias and were selected sequentially. It was presumed that the matched control participants were assessed simultaneously with their corresponding tonsillectomy participants (index date). Control participants who had deceased prior to the index date were excluded from the study. Throughout this matching process, 508,421 control participants were eliminated from consideration. Ultimately, 1240 tonsillectomy participants were successfully matched with 4960 control participants (Figure 1).

### 2.3. Definition of Tonsillectomy (Independent Variable)

Tonsillectomy was defined if the participants were treated by the medical claim code Q2300. Participants who had undergone tonsillectomy specifically for psoriasis were excluded from this categorization.

### 2.4. Definition of Psoriasis

Psoriasis was defined in participants diagnosed with the ICD-10 code L40. Only those who received treatment on more than two occasions were included in the study.

### 2.5. Covariates

Age was categorized into ten 5-year intervals, ranging from 40 to 44 years up to 85+ years. Income levels were divided into five classes, from class 1 (lowest income) to class 5 (highest income). The region of residence was classified as urban (Seoul, Busan, Daegu, Incheon, Gwangju, Daejeon, and Ulsan) or rural (Gyeonggi, Gangwon, Chungcheongbuk, Chungcheongnam, Jeollabuk, Jeollanam, Gyeongsangbuk, Gyeongsangnam, and Jeju).

Tobacco smoking status was categorized as nonsmoker, past smoker, or current smoker. Alcohol consumption was divided into less than once per week and once per week or more. Obesity was assessed using BMI (kg/m^2^), categorized according to the Asia–Pacific criteria from the Western Pacific Regional Office 2000 into underweight (<18.5), normal (≥18.5 to <23), overweight (≥23 to <25) obese I (≥25 to <30), and obese II (≥30) [17]. Systolic blood pressure (SBP, mmHg), diastolic blood pressure (DBP, mmHg), fasting blood glucose (mg/dL), and total cholesterol (mg/dL) were also measured.

The Charlson Comorbidity Index (CCI) was utilized to assess disease burden using 17 comorbidities, assigning scores based on severity and number of diseases. Connective tissue disorder was excluded from the CCI score in this study. CCI was treated as a continuous variable ranging from 0 (no comorbidities) to 29 (multiple comorbidities) [18,19].

In terms of outcomes, systemic lupus erythematosus (SLE, ICD-10 code: M32), ankylosing spondylitis (ICD-10 code: M45), rheumatoid arthritis (RA, ICD-10 code: M05, M06), and Sjogren syndrome (ICD-10 code: M350) were considered if treated more than twice. A history of steroid use was noted if there was at least one prescription.

### 2.6. Statistical Analyses

Propensity score overlap weighting was implemented to ensure covariate balance and an effective sample size. Propensity scores (PSs) were calculated using multivariable logistic regression incorporating all covariates. Overlap weighting, ranging from 0 to 1, was used to achieve a precise balance between groups. The standardized differences (SDs) before and after weighting were compared to assess disparities in general characteristics between tonsillectomy and control groups.

The overlap-weighted hazard ratios (HRs) for tonsillectomy on psoriasis were analyzed using a Cox proportional hazard regression model adjusted for variables such as age, sex, income, region of residence, obesity, smoking status, alcohol consumption, SBP, DBP, total cholesterol, CCI scores, asthma, systemic lupus erythematosus, ankylosing spondylitis, rheumatoid arthritis, and Sjogren syndrome.

Subgroup analyses were performed according to age (<50 years old and ≥50 years old), sex (male and female), income (low and high), and region of residence (urban and rural residents) using the overlap-weighted Cox proportional hazard regression model. Kaplan–Meier analysis was conducted, and the 95% confidence interval (CI) was calculated.

All analyses were two-tailed, and significance was determined at *p*-values less than 0.05. Statistical analyses were conducted using SAS version 9.4 (SAS Institute Inc., Cary, NC, USA).

## 3. Results

Table 1 outlines the general characteristics of the study participants. Within the tonsillectomy group, 1.37% had a documented history of psoriasis, compared to 1.11% in the control group. There were no significant differences between the matched participants in terms of age, sex, income, and region of residence, as indicated by an SD of 0.00 for these variables. However, disparities were observed in the distributions of obesity, smoking status, alcohol consumption, SBP, DBP, fasting blood glucose, total cholesterol, CCI scores, and the prevalence of SLE, ankylosing spondylitis, RA, Sjogren syndrome, and history of steroid use, with all SD values exceeding 0.01. After applying overlap weighting adjustments, all variables exhibited an SD of 0.00, indicating no significant differences between the two groups, with psoriasis rates of 1.30% in the tonsillectomy group and 1.20% in the control group.

The incidence rates of psoriasis were 15.4 per 10,000 person-years in the tonsillectomy group and 12.4 per 10,000 person-years in the control group (Table 2). The HR for psoriasis was 1.08 (95% CI = 0.69–1.69) among patients who underwent tonsillectomy compared to those without a history of tonsillectomy, after adjusting for factors such as obesity, smoking status, alcohol consumption, SBP, DBP, fasting blood glucose, total cholesterol, CCI scores, SLE, ankylosing spondylitis, RA, Sjogren syndrome, and history of steroid use. These differences were not statistically significant (*p* = 0.732).

The subgroup analyses by age, sex, income, and region of residency (Table 2) also showed no significant difference between two groups in any of the analyses (all *p* > 0.05). The adjusted HRs were 1.05 (95% CI = 0.62–1.76) in patients aged under 50 years old, 1.15 (95% CI = 0.45–2.91) in those aged 50 and older, 1.25 (95% CI = 0.76–2.06) in men, 0.57 (95% CI = 0.19–1.70) in women, 1.04 (95% CI = 0.60–1.81) in those with high income, 1.27 (95% CI = 0.58–2.76) in those with low income, 1.02 (95% CI = 0.53–1.98) in urban residents, and 1.18 (95% CI = 0.63–2.21) in rural residents.

**Figure 2:**
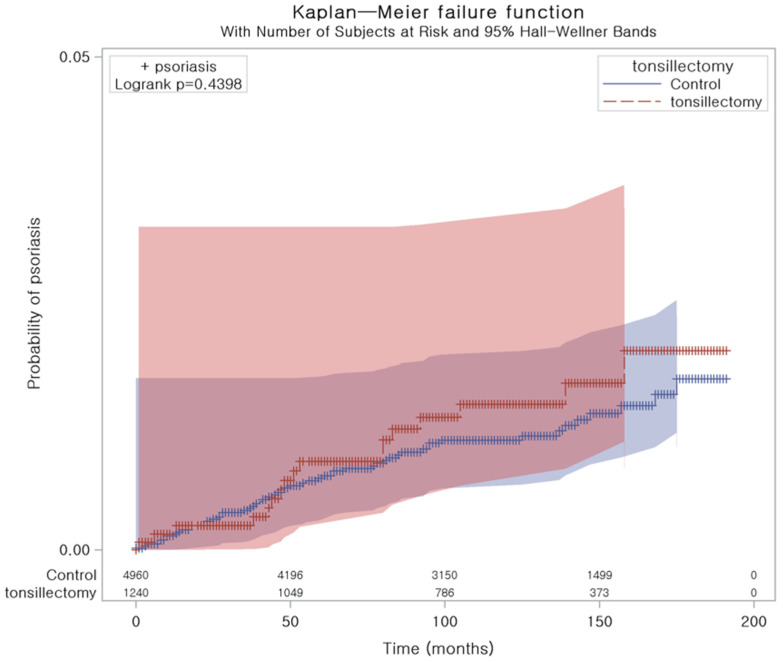
Kaplan–Meier curves of psoriasis in patients who underwent tonsillectomy.

Figure 2 illustrates the cumulative probability of psoriasis, which did not differ significantly between the tonsillectomy and control groups (Log-rank test: *p* = 0.440).

## 4. Discussion

The current study found no significant difference in the incidence rate and HR of late-onset psoriasis among patients who underwent tonsillectomy compared to control participants. Additionally, subgroup analyses failed to reveal any significant association between tonsillectomy and psoriasis.

Contrary to our results, a cohort study from Taiwan reported a decreased risk of psoriasis (adjusted HR = 0.43, 95% CI = 0.22–0.87) in the tonsillectomy group compared to the reference group [20]. A notable difference is that the Taiwanese study primarily included younger adults, mostly under 50 years of age. The strength of our study lies in its focus on late-onset, noninheritable types of psoriasis (>40 years of age), potentially minimizing genetic influences and more directly assessing the preventive impact of tonsillectomy. According to the Global Burden of Disease (GBD) 2019, the psoriasis burden increases notably in the 60–64 and 65–69 year age groups [1], highlighting the relevance of analyzing risks in an older population. Moreover, our definition of the psoriasis group—participants treated for psoriasis more than twice—enhances the reliability of our results. Analyses were adjusted for possible confounders such as smoking [18] and alcohol use [19].

There has been growing evidence suggesting that tonsillectomy might influence the development or exacerbation of psoriasis in certain individuals [8,10,21]. Psoriasis is often associated with an abnormal immune response, where T cells play a critical role in inflammation and skin cell turnover. The hypothesized mechanism involves the immune response to streptococcal infections, frequently impacting the tonsils and engaging psoriasis-related immune pathways. Specifically, the tonsils may act as a chronic source of streptococcal antigens, thus maintaining a persistent immune trigger that could exacerbate psoriasis [22]. For some, removing the tonsils might reduce this antigenic load and consequent immune activation, potentially diminishing the severity or incidence of psoriasis flares. However, outcomes vary widely among psoriasis patients. Additionally, because most studies are case reports or case series, the strength of evidence is limited. The first randomized, controlled clinical trial of tonsillectomy in 29 patients with plaque psoriasis showed a significant benefit of tonsillectomy compared to control group [21]. After tonsillectomy, 13 of 15 patients (86%) experienced improvement in psoriasis symptoms, ranging from a 30% to 90% decrease in their PASI scores. Furthermore, they showed a decrease in peptide-reactive skin-homing T cells in their peripheral blood; this decrease did not occur in the control group. However, the complete pathophysiology is still not well understood and further research is still needed.

There are several reasons why tonsillectomy may not prevent late-onset psoriasis effectively. Participants who underwent tonsillectomy in this study might be non-responders. A systematic review showed that while some patients experienced sustained improvement in psoriasis, others experienced psoriasis relapse after tonsillectomy [10]. The variability could be linked to the incomplete elimination of peptide-reactive skin-homing T cells from the blood, as streptococci may persist in lymphatic tissues other than tonsils. Additionally, tonsillectomy may not significantly reduce streptococcal infections. A previous study suggested that tonsillectomy might increase the risk of retropharyngeal and parapharyngeal abscess in adults but not in children [23]. A Cochrane review concluded that sufficient evidence does not exist to indicate that a tonsillectomy changes the number sore throat episodes [24]. While tonsillectomy might alleviate symptoms and manage existing psoriasis, its preventive efficacy remains uncertain. The development of psoriasis is complex and influenced by genetic and environmental factors. The lack of association between tonsillectomy and psoriasis may partly arise because tonsillectomy does not alter predispositions which are significant contributors to psoriasis. A previous study reported that HLA-Cw0602 was found in 69.6% of Korean psoriasis patients [3]. Moreover, environmental triggers other than those potentially mitigated by tonsillectomy such as skin trauma, stress, or medication may play a more prominent role in the onset of psoriasis. A cross-sectional study identified whether exacerbating factors in psoriasis differed by ethnic groups, and found that infection was a less common trigger and stress was a common cause for psoriasis in Asian individuals [25].

The results of the present study, which found that tonsillectomy does not have a preventive effect for psoriasis, are important because they may be beneficial to clinicians managing patients with psoriasis and otorhinolaryngologists. It is important to note that tonsillectomy carries risks and potential complications, such as bleeding, infection, and anesthetic complications. Therefore, the decision to perform a tonsillectomy for the potential prevention of psoriasis should be carefully considered, taking into account the severity and frequency of the tonsillitis. Further longitudinal studies and randomized controlled trials are needed to establish a more robust evidence base that can guide clinical decisions regarding the use of tonsillectomy as a preventive treatment for psoriasis.

Some limitations warrant consideration. First, this study utilized a retrospective electronic database, which is subject to potential misclassification and inaccuracies in diagnostic coding. To mitigate this issue, only participants who received treatment for psoriasis more than twice were classified within the psoriasis group. Second, the indications for tonsillectomy were not explicitly confirmed, and it is uncertain whether the procedure was performed due to tonsillitis. However, it is well-documented that tonsillectomy is predominantly conducted to address the infectious complication of tonsillitis, and previous studies have indicated that treatment for recurrent infections or chronic inflammation is the most common reason for tonsillectomy across all age groups [26]. Third, since information on different types of psoriasis, such as guttate, inverse, pustular, erythrodermic, or plaque, was unavailable in our dataset, types of psoriasis were not specifically categorized and were included into one group overall. As different types of psoriasis may be caused by various etiologies and result in different complications, further studies conducted according to each type of psoriasis should be performed to overcome the effect of heterogenous features of psoriasis. Fourth, our dataset did not allow for the measurement of lymphocyte levels. Future molecular studies are essential to provide insights into lymphocyte changes. Lastly, this study population was restricted to individuals aged 40 or older. Although a previous Korean epidemiologic study identified the highest prevalence of psoriasis in individuals in their 40s and 50s [3], this focus enabled us to specifically examine the incidence of late-onset psoriasis after tonsillectomy.

## 5. Conclusions

In the Koran adult population, late-onset psoriasis showed no association with a history of tonsillectomy. Future longitudinal studies with extended follow-up periods are also needed to assess whether tonsillectomy performed in childhood could influence the development of psoriasis later in adulthood. This nuanced understanding also highlights the complexity of psoriasis treatment and prevention, indicating the need for further research to clarify the role of tonsillectomy in managing this challenging skin disorder.

## Figures and Tables

**Table 1:** General characteristics of participants.

Characteristics	Tonsillectomy	Control	Standardized Difference
Age (years old) (*n*, %)			0.00
40–44	74 (5.97)	296 (5.97)	
45–49	248 (20.00)	992 (20.00)	
50–54	336 (27.10)	1344 (27.10)	
55–59	315 (25.40)	1260 (25.40)	
60–64	153 (12.34)	612 (12.34)	
65–69	74 (5.97)	296 (5.97)	
70–74	25 (2.02)	100 (2.02)	
75–79	9 (0.73)	36 (0.73)	
80–84	5 (0.40)	20 (0.40)	
85+	1 (0.08)	4 (0.08)	
Sex (*n*, %)			0.00
Male	837 (67.50)	3348 (67.50)	
Female	403 (32.50)	1612 (32.50)	
Income (*n*, %)			0.00
1 (lowest)	141 (11.37)	564 (11.37)	
2	123 (9.92)	492 (9.92)	
3	153 (12.34)	612 (12.34)	
4	256 (20.65)	1024 (20.65)	
5 (highest)	567 (45.73)	2268 (45.73)	
Region of residence (*n*, %)			0.00
Urban	623 (50.24)	2492 (50.24)	
Rural	617 (49.76)	2468 (49.76)	
Obesity (*n*, %) *			0.31
Underweight	7 (0.56)	101 (2.04)	
Normal	286 (23.06)	1630 (32.86)	
Overweight	346 (27.90)	1433 (28.89)	
Obese I	530 (42.74)	1661 (33.49)	
Obese II	71 (5.73)	135 (2.72)	
Smoking status (*n*, %)			0.08
Nonsmoker	721 (58.15)	2939 (59.25)	
Past smoker	247 (19.92)	836 (16.85)	
Current smoker	272 (21.94)	1185 (23.89)	
Alcohol consumption (*n*, %)			0.04
<1 time a week	720 (58.06)	2780 (56.05)	
≥1 time a week	520 (41.94)	2180 (43.95)	
SBP (*n*, %)	125.33 (14.33)	124.49 (15.59)	0.06
DBP (*n*, %)	78.79 (10.48)	78.19 (10.58)	0.06
Fasting blood glucose (*n*, %)	99.00 (22.26)	99.85 (27.45)	0.03
Total cholesterol (*n*, %)	198.20 (36.73)	198.66 (35.93)	0.01
CCI score (mean, SD)	0.90 (1.61)	0.64 (1.32)	0.17
Systemic lupus erythematosus (*n*, %)	0 (0.00)	7 (0.14)	0.05
Ankylosing spondylitis (*n*, %)	13 (1.05)	36 (0.73)	0.03
Rheumatoid arthritis (*n*, %)	136 (10.97)	411 (8.29)	0.09
Sjogren syndrome (*n*, %)	4 (0.32)	18 (0.36)	0.01
Steroid (*n*, %)	1234 (99.52)	4701 (94.78)	0.29
Psoriasis (*n*, %)	17 (1.37)	55 (1.11)	0.02

* Obesity (BMI, body mass index, kg/m^2^) was categorized as <18.5 (underweight), ≥18.5 to <23 (normal), ≥23 to <25 (overweight), ≥25 to <30 (obese I), and ≥30 (obese II). CCI, Charlson Comorbidity Index; SBP, systolic blood pressure; and DBP, diastolic blood pressure.

**Table 2:** Crude and adjusted hazard ratios (95% confidence interval) of tonsillectomy group compared to control group for psoriasis with stratified subgroup according to age, sex, income, and region of residence.

Independent Variables		Psoriasis/ Participants (*n*, %)	Follow-Up Duration (PY)	IR per 10,000 (PY)	Hazard Ratios (95% CI) for Psoriasis			
					Crude ^†^	*p*-Value	Adjusted ^†,‡^	*p*-Value
Total participants (*n* = 6200)								
	Tonsillectomy	17/1240 (1.4)	11,060	15.4	1.24 (0.72–2.13)	0.441	1.08 (0.69–1.69)	0.732
	Control	55/4960 (1.1)	44,325	12.4	1		1	
Age < 50 years old (*n* = 4865)								
	Tonsillectomy	13/973 (1.3)	9426	13.8	1.24 (0.66–2.30)	0.505	1.05 (0.62–1.76)	0.866
	Control	42/3892 (1.1)	37,663	11.2	1		1	
Age ≥ 50 years old (*n* = 1335)								
	Tonsillectomy	4/267 (1.5)	1634	24.5	1.27 (0.41–3.89)	0.679	1.15 (0.45–2.91)	0.774
	Control	13/1068 (1.2)	6662	19.5	1		1	
Men (*n* = 4185)								
	Tonsillectomy	15/837 (1.8)	7593	19.8	1.43 (0.80–2.58)	0.231	1.25 (0.76–2.06)	0.389
	Control	42/3348 (1.3)	30,451	13.8	1		1	
Women (*n* = 2015)								
	Tonsillectomy	2/403 (0.5)	3467	5.8	0.61 (0.14–2.72)	0.521	0.57 (0.19–1.70)	0.314
	Control	13/1612 (0.8)	13,874	9.4	1		1	
High income (*n* = 4115)								
	Tonsillectomy	11/823 (1.3)	3611	30.5	1.16 (0.59–2.27)	0.470	1.04 (0.60–1.81)	0.886
	Control	38/3292 (1.2)	14,395	26.4	1		1	
Low income (*n* = 2085)								
	Tonsillectomy	6/417 (1.4)	7449	8.1	1.41 (0.56–3.57)	0.662	1.27 (0.58–2.76)	0.556
	Control	17/1668 (1.0)	29,930	5.7	1		1	
Urban residents (*n* = 2630)								
	Tonsillectomy	7/623 (1.1)	5629	12.4	1.07 (0.47–2.47)	0.870	1.02 (0.53–1.98)	0.950
	Control	26/2492 (1.0)	22,371	11.6	1		1	
Rural residents (*n* = 2780)								
	Tonsillectomy	10/617 (1.6)	5431	18.4	1.39 (0.68–2.86)	0.0366	1.18 (0.63–2.21)	0.601
	Control	29/2468 (1.2)	21,954	13.2	1		1	

^†^ Models were stratified by age, sex, income, and region of residence. ^‡^ The model was adjusted for obesity, smoking, alcohol consumption, systolic blood pressure, diastolic blood pressure, fasting blood glucose, total cholesterol, CCI scores, systemic lupus erythematosus, ankylosing spondylitis, rheumatoid arthritis, Sjogren syndrome, and history of steroid use.

## Data Availability

Data in this study were from the Korean National Health Insurance Service-Health Screening Cohort. Release of the data by the researcher is not legally allowed. All data are available from the database of the National Health Insurance Sharing Service (NHISS) (https://nhiss.nhis.or.kr/) (accessed on 10 January 2022). The NHISS allows access to all data for any researcher who promises to follow the research ethics at some cost. If you want to access the data of this article, you can download it from the website after promising to follow the research ethics.
